# Alginate Gel Reinforcement with Chitin Nanowhiskers Modulates Rheological Properties and Drug Release Profile

**DOI:** 10.3390/biom9070291

**Published:** 2019-07-19

**Authors:** Valentina A. Petrova, Vladimir Y. Elokhovskiy, Sergei V. Raik, Daria N. Poshina, Dmitry P. Romanov, Yury A. Skorik

**Affiliations:** 1 Institute of Macromolecular Compounds of the Russian Academy of Sciences, Bolshoy pr. V.O. 31, St Petersburg 199004, Russia; 2 Institute of Silicate Chemistry of the Russian Academy of Sciences, Adm. Makarova emb. 2, St. Petersburg 199034, Russia; 3 Almazov National Medical Research Centre, Akkuratova str. 2., St. Petersburg 197341, Russia

**Keywords:** alginic acid, chitin, hydrogel, tetracycline, drug delivery

## Abstract

Hydrogels are promising materials for various applications, including drug delivery, tissue engineering, and wastewater treatment. In this work, we designed an alginate (ALG) hydrogel containing partially deacetylated chitin nanowhiskers (CNW) as a filler. Gelation in the system occurred by both the protonation of alginic acid and the formation of a polyelectrolyte complex with deacetylated CNW surface chains. Morphological changes in the gel manifested as a honeycomb structure in the freeze-dried gel, unlike the layered structure of an ALG gel. Disturbance of the structural orientation of the gels by the introduction of CNW was also expressed as a decrease in the intensity of X-ray diffraction reflexes. All studied systems were non-Newtonian liquids that violated the Cox-Merz rule. An increase in the content of CNW in the ALG-CNW hydrogel resulted in increases in the yield stress, maximum Newtonian viscosity, and relaxation time. Inclusion of CNW prolonged the release of tetracycline due to changes in diffusion. The first phases (0–5 h) of the release profiles were well described by the Higuchi model. ALG-CNW hydrogels may be of interest as soft gels for controlled topical or intestinal drug delivery.

## 1. Introduction

Hydrogels based on natural polysaccharides are promising materials for biomedical applications due to their biocompatibility, biodegradability, and wide range of physical properties [1]. Polysaccharides can be used in the form of films, sponges, and hydrogels for purposes that include wound-healing and burn coatings [2,3], tissue engineering [4,5], drug and growth factor delivery [6], and suturing [7].

Hydrogels and composite materials based on the natural polysaccharide alginic acid (ALG) are well known and widely used in bone tissue engineering [8], drug delivery [9], and cell encapsulation [10,11]. ALG molecules are linear and contain β-d-mannuronic and α-l-guluronic acid residues that are present in their pyranose forms and are linked by 1–4 bonds. Ionotropic ALG gels are obtained by adding multiply charged cations (e.g., Ca^2+^, Ba^2+^, Cu^2+^, Al^3+^), which act as crosslinking agents. These cations interact with the carboxylic groups of the guluronate units of the polysaccharide molecules, whereas the mannuronate units remain free [12]. ALG does not form ionotropic gels if the mole fraction of guluronic acid in the polysaccharide is less than 20–25% [13]. In Reference [14], an electrodialysis method for the preparation of ALG gels crosslinked with Ca^2+^ was described. Modulation of the conditions for electrodialysis created variations in the degree of gel crosslinking. Homogeneous gels can be created by the method of delayed gelling of ALG [15], which is accomplished by the decomposition of calcium carbonate with slow acidification by hydrolysis of D-glucono-1,5-lactone.

The polyanionic nature of ALG allows it to interact electrostatically with polycations to form polyelectrolyte complexes (PEC) [16,17]. The formation and stability of these PEC depend on numerous factors, including the degree of ionization, charge density, and molecular weight of the polymers; the nature and position of ionogenic groups; the flexibility of the polymer chains; the concentration and order of mixing of the polyelectrolytes during the formation of the PEC; and the temperature, ionic strength, and pH of the medium [18]. This ability to form PEC increases the attractiveness of ALG as a hydrogel component.

Recently, much attention has been paid to composite hydrogels that show a combination of the properties of their individual components [19]. The characteristics of a composite hydrogel are determined by the physicochemical properties of its components and by the structure of the material. Various hydrogel structures are possible, ranging from structures with a complete separation of polymer phases to those comprising a matrix with nanoscale inclusions or with continuous phases of both polymers. The biphasic nature of composite hydrogels, as a rule, determines their advantages when used for purposes that include superabsorbents, membrane materials, substitutes for living tissues, carriers of medicinal substances, and materials for making soft contact lenses. Practically all known methods can be used to produce composite hydrogels based on hydrophilic polymers; one simple example is the combination of polymers in solution and their binding as a result of various physical and chemical interactions [20,21].

Several systems based on ALG/chitosan ionic interactions have been studied. PEC hydrogels used for drug delivery are usually in the form of microparticles or beads. For example, Sarmento et al. [22] revealed that anionic PEC particles can provide prolonged insulin release and can increase oral insulin bioavailability. Applications of chitosan and ALG systems for protein and peptide delivery have been described in a review [23]. Layer-by-layer coating of ALG hydrogel with chitosan has provided a vehicle for intestinal delivery of probiotics [24]. Moreover, an ALG gel containing crosslinked chitosan has shown promise for Hg^2+^ removal from water solutions [25].

The use of nanofillers consisting of polysaccharides with a fibrillar structure, such as chitin and cellulose, can lead to interesting properties of the composite materials [26,27,28]. For instance, chitin nanocrystals have been incorporated into supramolecular cyclodextrin-based hydrogels as a way to increase the mechanical strength of the hydrogel and its capability for controlled drug release [29]. The presence of polysaccharide nanocrystals increases the stability of the hydrogel structure and provides a stable release profile of a biologically active substance without causing additional cytotoxicity, compared with the original hydrogel.

Chitin nanowhiskers (CNW) have been used as fillers in the preparation of ALG microcapsules crosslinked with Ca^2+^ ions [30]. Partially deacetylated CNW contain positively charged amino groups and are also capable of interacting with negatively charged carboxyl groups of ALG to form PEC. Thus, CNW can act as natural crosslinking agents that can change the structure and stability of an ALG hydrogel, alter the mechanical properties, and modulate the controlled release of drugs. Moreover, the rigid chitin core provides a defined structure for the nanoparticle, while deacetylated chains on the surface can be chemically modified [31].

The controlled release of drugs or other biologically active substances can solve problems that arise in situations where a constant concentration of a therapeutically active compound is needed in the blood, where a predictable rate of release is required over a long period of time, or where an unstable bioactive compound must be protected [32]. Antibiotics are a class of drugs that present particular challenges for controlled release, and the development of antibiotic formulations in the form of hydrophilic gels has been particularly problematic. Typically, antibiotics (e.g., tetracycline and erythromycin) are applied as ointments composed of a classic hydrophobic base, usually a mixture of petroleum jelly and lanolin. However, these ointment bases have their drawbacks, primarily that they are susceptible to contamination by pathogenic microorganisms. Hydrophilic bases, including cellulose derivatives (e.g., methylcellulose, sodium carboxymethylcellulose, etc.) and, more rarely, crosslinked polymers of acrylic acid or ALG, can also be used as carriers for antibiotics. These bases are resistant to microbial contamination, are nontoxic, and do not trigger allergic reactions [33,34].

The purpose of the present work was to obtain hydrogels based on ALG and partially deacetylated CNW. We hypothesized that cationic CNW may act as an active nanofiller that can change the hydrogel structure. Therefore, we investigated the rheological and structural properties of ALG-CNW hydrogels and their influence on the tetracycline release profile. The obtained results may be useful for both topical and intestinal tetracycline delivery.

## 2. Materials and Methods

### 2.1. Materials

In this work, we used sodium alginate with a molecular weight (MW) of 1.3 × 10^5^ (Qingdao Bright Moon Seaweed Group Co. LTD, China).

CNWs were obtained by partial deacetylation of α-chitin with a particle size of 0.1–0.2 mm, as previously reported [30,31]. The degree of deacetylation (DDA) was determined by conductometric titration (DDA 0.40 ± 0.03) and by elemental analysis (DDA 0.40 ± 0.02) [31]. The size of the CNWs (thickness 6–15 nm, length 100–500 nm) was estimated by scanning electron microscopy [31].

Tetracycline hydrochloride was provided by JSC Vertex (St. Petersburg, Russia). Other reagents and solvents were of reagent grade and were used without further purification.

### 2.2. Preparation of ALG-CNW Hydrogels

To obtain ALG-CNW hydrogels, concentrated solutions of ALG were mixed with a 0.5% CNW aqueous dispersion, and the prescribed amount of water was then added to obtain 4% ALG solution. The resulting ALG-CNW dispersion (10 g) was homogenized for 1 h with mechanical stirring, followed by addition of 0.2 mL 2% acetic acid solution under vigorous stirring. Hydrogels were obtained with an ALG concentration of 4% and a mass fraction of CNW of 0, 2.5, 7.5, and 14.5% relative to ALG. The pH values of the obtained hydrogels were 4.3 ± 0.2. The hydrogels were kept at room temperature for 1 day and then stored in a refrigerator at 4 °C for 2 days.

Hydrogels containing tetracycline were obtained by mixing an aqueous solution of tetracycline and ALG. First, 60 mg of tetracycline was dissolved in water and mixed with the ALG solution. This mixture was then mixed as described above to obtain the ALG-CNW hydrogels. The final tetracycline concentration in the gel was 1.5 mg/g.

### 2.3. Isolation of ALG-CNW Microgels

To isolate the ALG-CNW microgels, the ALG-CNW hydrogel was diluted with water to destroy the physically linked gel. The microgels were separated from the ALG solution by centrifugation (MPW-380R, Poland) at 4500 rpm, washed with water, and freeze-dried. For measurements of the hydrodynamic radii and ζ-potential, the microgels were redispersed in water at 1 mg/mL and then stirred for 24 h. The large aggregates were then separated by centrifugation (2 min, 2000 rpm) and the suspension was diluted to 0.1 mg/mL.

### 2.4. General Methods

The rheological properties of the hydrogels were studied at 25 °C with a Physica MCR301 rheometer (Anton Paar GmbH, Graz, Austria) in a CP25-2 cone-plate measuring system for the shear and oscillatory tests.

X-ray diffraction analysis was performed with a DRON-3M (Burevestnik, St. Petersburg, Russia) instrument using Ni-filtered Cu Kα radiation (λ = 1.5418 Å).

The surface morphology was captured by reflected light at 100× magnification using a Levenhuk D870T optical microscope (Levenhuk Ltd., Long Island City, NY, USA) equipped with a digital camera.

The hydrodynamic radii and ζ-potential of the ALG-CNW microgels and CNW were measured with a Photocor Compact-Z device (Photocor Ltd., Moscow, Russia) with a 659.7 nm He–Ne laser at 25 mV power and a detection angle of 90°.

Elemental analysis was performed using a Vario Micro Cube analyzer (Elementar Analysensysteme GmbH, Langenselbold, Germany).

### 2.5. Tetracycline Release Kinetics

The release of tetracycline from hydrogels containing tetracycline was determined using the following procedure: 1 g of a gel containing 1.5 mg of tetracycline was placed in a plastic tube with a dialysis membrane fixed to the end and the tetracycline-containing gel evenly distributed over the surface of the dialysis membrane. The tube was immersed in a vessel containing 30 mL saline (0.9% NaCl solution). The release was promoted by constant stirring at 30 °C. At specific time intervals, 1 mL of the solution was removed, combined with 0.3 mL 1 M NaOH, and used to determine the concentration of tetracycline spectrophotometrically with an Ocean Optics USB4000 spectrophotometer (Ocean Optics Inc., Largo, FL, USA) using a calibration curve (380 nm, 0–0.05 mg/mL; R^2^ = 0.998). The sampled volume was replaced with 1 mL of saline.

## 3. Results and Discussion

### 3.1. Preparation of ALG-CNW Hydrogels

The formation of the ALG-CNW hydrogel started immediately upon acidification. A slow increase in viscosity was observed until the formation of a hydrogel capable of keeping its shape. Presumably, the gel-forming centers in the ALG-CNW hydrogels were the positively charged CNW, which are capable of forming PEC with negatively charged ALG molecules. Further gelation is associated with the formation of a physical ALG gel, possibly due to the conversion of a part of the ALG to the protonated form or due to the formation of hydrogen bonds between the hydroxyl and carboxyl groups of the pyranose rings of L-guluronic acid in neighboring polymer chains. The primary action that leads to the formation of physical gels is molecular entanglements, in addition to ionic and hydrogen bonding and hydrophobic interactions.

**Figure 1:**
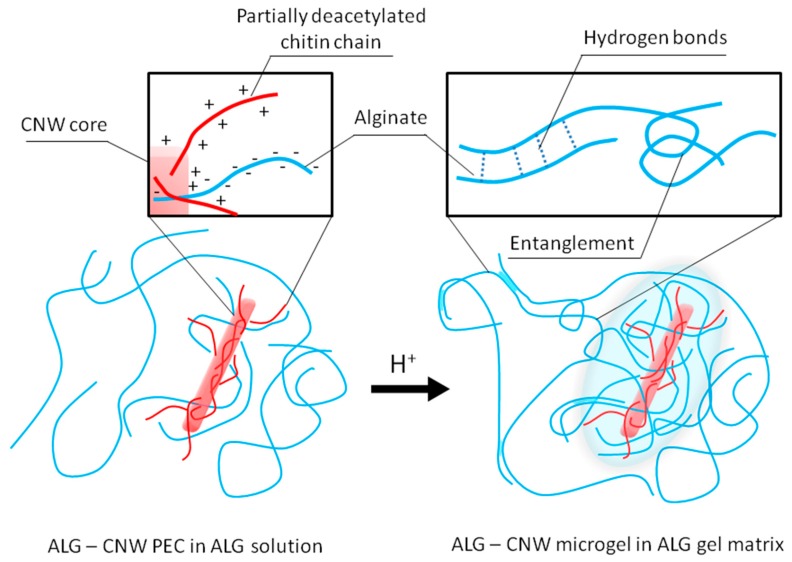
Scheme of the formation of alginate (ALG) hydrogel containing partially deacetylated chitin nanowhiskers (CNW).

We believe that the ALG-CNW hydrogels are formed both by electrostatic interaction (i.e., formation of a PEC due to the interaction of the positively charged amino groups of CNW and the negatively charged carboxyl groups of ALG) and by other physical interactions (molecular entanglements of ALG chains, hydrogen bonding) and thus represent a two-phase system. When water is added to the hydrogel, the physical gel is slowly destroyed, while the main part of the ALG goes into solution (Figure 1).

The molar ratio between the monomeric units of ALG and CNW in the microgels can be estimated using the elemental analysis data and the following equation:

(1) $$\left[ALG\right]:\left[CNW\right]=\frac{1}{x}\left[{\left(\frac{\omega_{C}}{\omega_{N}}\right)}_{ALG-CNW}-{\left(\frac{\omega_{C}}{\omega_{N}}\right)}_{CNW}\right]\frac{MW_{N}}{MW_{C}}=3.2,$$

where *x* is the number of C atoms in the ALG monomeric units (*x* = 6); *ω* is the mass fraction of the corresponding element (CNW: C 43.09%, N 6.98%; ALG-CNW microgels: C 36.08%, N 1.60%); and *MW* is the corresponding molecular weight.

Elemental analysis showed that the microgels represent a PEC formed between CNW and ALG (with a triple excess of ALG). Unlike the positively charged CNW (ζ potential +20 ± 2 mV) with R_h_ of 300 ± 10 nm, the microgels isolated from the ALG-CNW hydrogel had a negative ζ-potential of -51 ± 1.7 mV and R_h_ of 725 ± 60 nm (pH of the microgel dispersion was 5.0). Particles with ζ-potential of more than 30 mV (either positive or negative) are usually considered stable.

### 3.2. Structure and Morphology of Hydrogels

**Figure 2:**
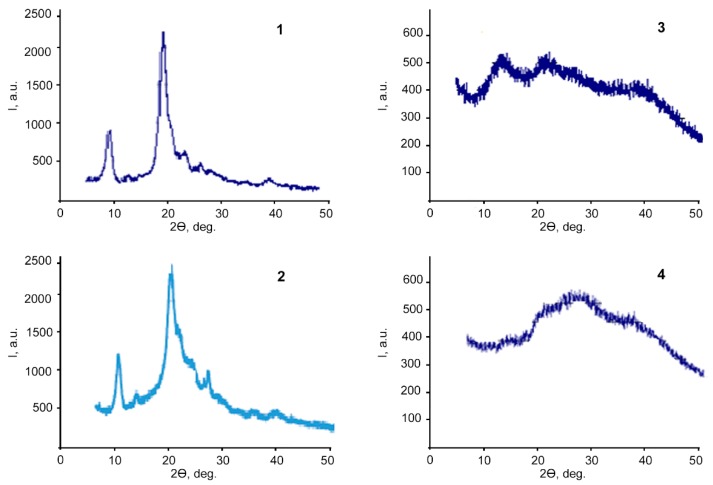
X-ray diffraction patterns: 1—CNW; 2—lyophilized ALG-CNW microgel, isolated from the ALG-CNW (7.5%) hydrogel; 3—lyophilized ALG hydrogel; 4—lyophilized ALG-CNW (7.5%).

The X-ray diffractogram of the ALG-CNW microgels isolated from a hydrogel (Figure 2-2) indicated the retention of the structure of CNW (Figure 2-1), except for a signal broadening at 2θ = 23°.

**Figure 3:**
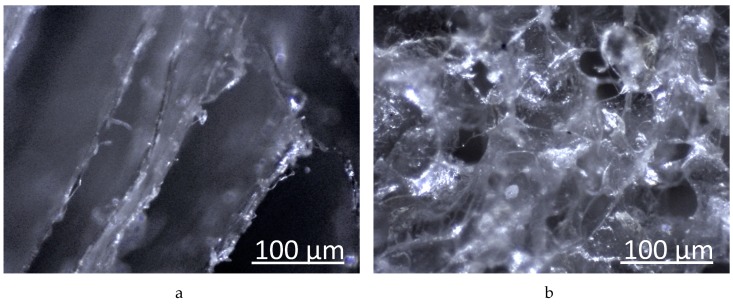
Micrographs (100×) of thin sections of freeze-dried hydrogels (**a**) ALG, (**b**) ALG-CNW (7.5%).

**Figure 4:**
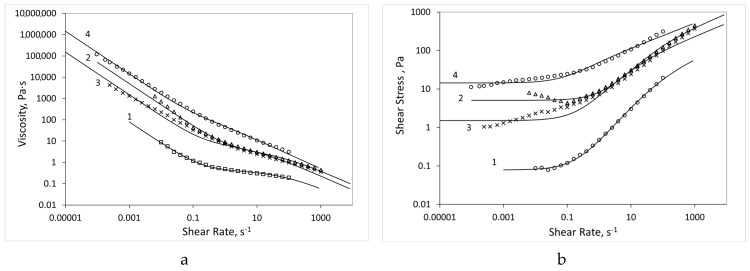
Dependence of viscosity (**a**) and shear stress (**b**) on the shear rate in the shear test (Down SR mode). Dots represent experimental values, lines were fitted using the Cross equation. 1—ALG, —ALG-CNW (2.5%), 3—ALG-CNW (7.5%), 4—ALG-CNW (14.5%).

The diffractogram of the lyophilized ALG hydrogel (Figure 2-3) had reflexes at 2θ = 13° and 23°, which is also characteristic of ALG itself. The diffractogram of the lyophilized ALG-CNW (7.5%) (Figure 2-4) was characterized by a significant decrease in the reflex at 2θ = 13° and a weakly pronounced reflex at 2θ = 23°, which are also characteristic of ALG. Thus, the analysis shows a different structure of the ALG-CNW and ALG hydrogels.

Examination of the surface morphology of thin sections of lyophilized hydrogels also revealed a different structural organization of the hydrogels (Figure 3). For the ALG hydrogel, we observed a layered structure, and for the ALG-CNW hydrogel, the structure was of the honeycomb type.

### 3.3. Rheological Properties of Hydrogels

The rheological properties of the hydrogels were studied by varying the content of CNW in the ALG gel in a range from 0 to 14.5% CNW (relative to ALG).

The rheological tests of the hydrogels were performed using shear testing with a decrease in the shear rate (Down SR mode) from 100 s^−1^ to the lowest possible value (usually 0.0001 s^−1^). A high shear rate destroys the structure of the gel, thereby eliminating the influence of the stressing history. In this test, a decrease in the shear rate results in a growth of the structure.

The dependence of viscosity and shear stress on the shear rate (Figure 4) indicates that all the tested compositions are non-Newtonian liquids with a structure characteristic of gels.

The shear test in the Top SR mode was carried out with an increase in the shear rate from the minimum to the maximum possible.

Dynamic measurements in the oscillatory mode were also conducted by decreasing the angular frequency from 100 to 0.1 rad/s (Down F mode) and by increasing from the minimum value of the circular frequency to 100 rad/s (Top F mode).

The shear test in the Down SR mode assumes the most destroyed gel structure, where the system behaves like a structured liquid (Figure 4) that can be described by the Cross equation with yield stress:

(2) $$\tau \left(\dot{\gamma }\right)=\tau_{0}+\eta_{\infty }\dot{\gamma }+\frac{\left(\eta_{0}-\eta_{\infty }\right)\dot{\gamma }}{1+{\left(\theta \dot{\gamma }\right)}^{p}}$$

(3) $$\eta \left(\dot{\gamma }\right)=\frac{\tau_{0}}{\dot{\gamma }}+\eta_{\infty }+\frac{\left(\eta_{0}-\eta_{\infty }\right)}{1+{\left(\theta \dot{\gamma }\right)}^{p}}$$

where $\tau \left(\dot{\gamma }\right)$ is the shear stress (Pa) as a function of shear rate (s^−1^); *τ_0_* is the yield stress; $\left(\dot{\gamma }\right)$, $\eta_{0}$, $\eta_{\infty }$ are the effective viscosity, maximum, and minimum Newtonian viscosity, respectively (Pa·s); θ is the relaxation time (s); *p* is the power index (for many polymers, this is equal to 2/3).

The contribution of the yield stress at high rates was not significant and appeared at low shear rates. The calculation was performed by varying the parameters with an accuracy of 1%; the calculation criterion was the minimum standard deviation (SD) of the viscosity. The lowest Newtonian viscosity is usually the viscosity of the solvent (in this case, 0.0009 Pa·s, which is the viscosity of the acetic acid solution at 25 °C).

**Figure 5:**
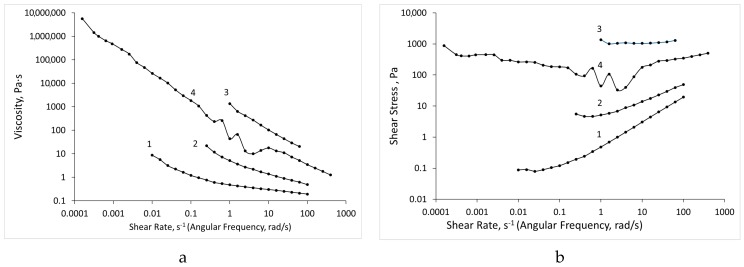
Dependence of viscosity (**a**) and shear stress (**b**) on the shear rate (angular frequency) of the ALG hydrogel: 1—shear test in the Down SR mode, 2—dynamic test in the Down F mode, 3—shear test in the Top SR mode, 4—dynamic test in the Top F mode.

**Figure 6:**
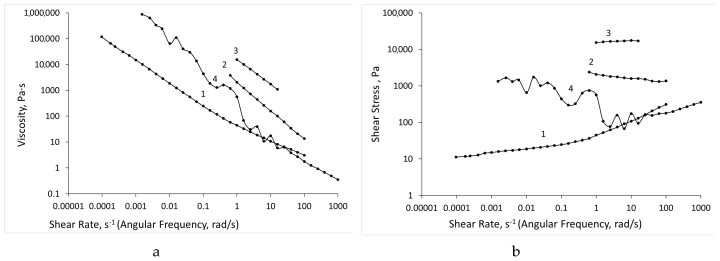
Dependence of viscosity (**a**) and shear stress (**b**) on the shear rate (angular frequency) of the ALG-CNW (7.5%) hydrogel: 1—shear test in the Down SR mode, 2—dynamic test in the Down F mode, 3—shear test in the Top SR mode, 4—dynamic test in the Top F mode.

The tests were carried out over time at a constant shear rate (or angular frequency); therefore, the gel was structured and the structure grew with time, regardless of the type of test (Figure 5 and Figure 6). At this point, the Cross formula no longer correctly described the system. This is especially well seen by the dependences of the shear stress on the shear rate (angular frequency), as shown in Figure 5 and Figure 6. The Cox-Merz rule (i.e., the dynamic viscosity is equal to the shear viscosity when the values of the angular frequency and shear rate are equal) did not hold for the studied systems.

In the shear test, the assembly and destruction of the gel structure occurred simultaneously (especially at high strain rates); therefore, the strength of the structure was somewhat lower than in the dynamic mode. All the ALG-CNW hydrogels shown in Figure 4 behaved similarly.

All the ALG-CNW compositions after the shear test (Down SR mode) went to the gel state, and no dependence on the shear rate (angular frequency) was observed. The shear stress induced a flow that depended on the history of stressing and varied over a wide range. ALG gels with CNW had a stronger structure, with a yield stress reaching 17,000 Pa for the ALG-CNW (14.5%); for the ALG gel, this value was lower than 1120 Pa. The effect of the structure on the viscosity was noticeable at strain rates below 0.001 s^−1^. The dynamic loss factor (dynamic loss tangent) ranged from 1 to 0.1, which is typical for the gel.

The rheological properties of the ALG-CNW hydrogels are summarized in Table 1.

**Figure 7:**
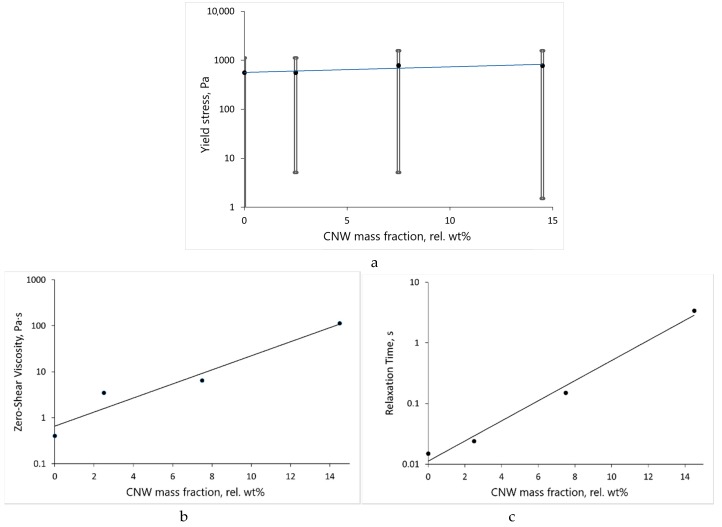
Dependencies of the yield stress (**a**), maximum Newtonian viscosity (**b**), and relaxation time (**c**) of hydrogels on the CNW content. For the yield stress, the minimum and maximum values are shown.

An increase in the content of CNW in the ALG-CNW hydrogel resulted in increases in the yield stress, maximum Newtonian viscosity, and relaxation time (Figure 7). The spread of the yield stress was almost equal to 100%; this is due to its constant growth during the testing process.

### 3.4. Release of Tetracycline from ALG-CNW Hydrogels

**Figure 8:**
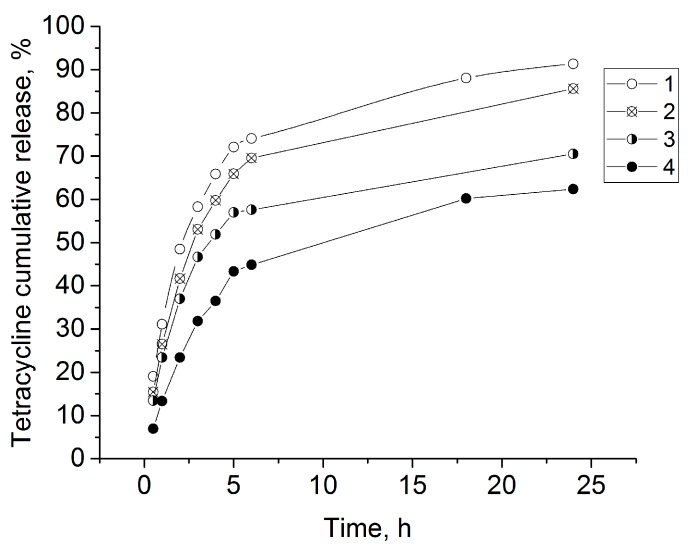
Tetracycline release kinetics from gels: 1—ALG, 2—ALG-CNW (2.5%), 3—ALG-CNW (7.5%), 4—ALG-CNW (14.5%).

Tetracycline was released more slowly from the ALG-CNW hydrogels than from the ALG gel (Figure 8).

**Figure 9:**
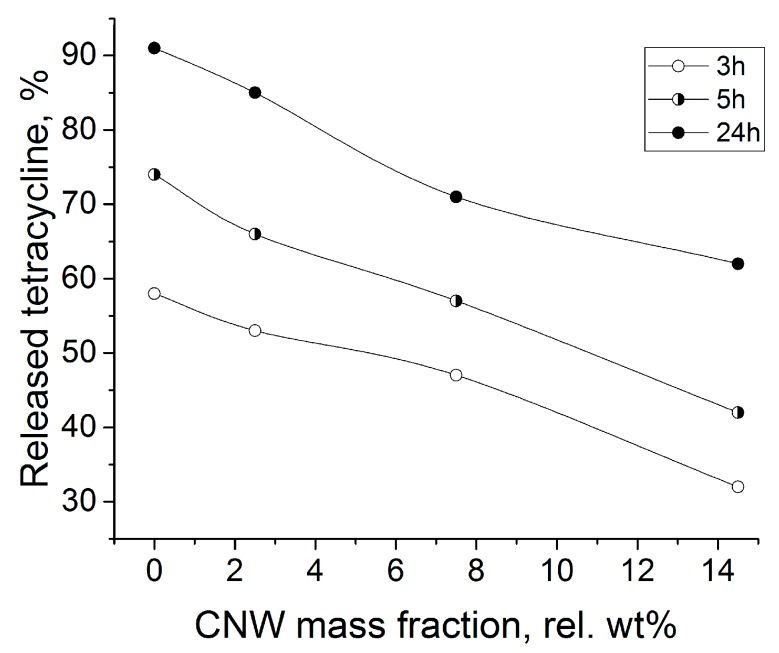
Dependence of the fraction of released tetracycline on the mass fraction of CNW at different release times.

For the ALG CNW hydrogels, a prolonged release of tetracycline was observed for 24 h and was dependent on the amount of CNW (Figure 9).

**Figure 10:**
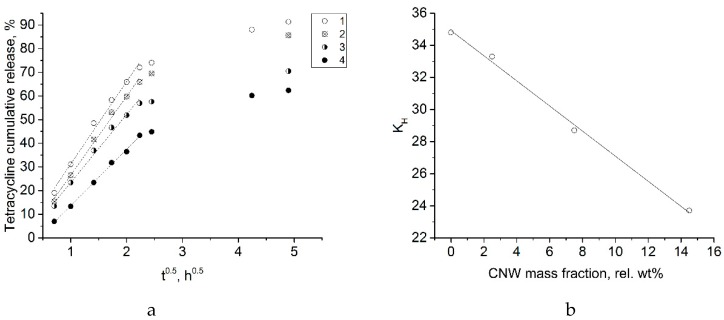
(**a**) Linearized tetracycline release curves plotted as a cumulative release vs. square root of time for ALG-CNW hydrogels: 1—ALG, 2—ALG-CNW (2.5%), 3—ALG-CNW (7.5%), 4—ALG-CNW (14.5%); (**b**) K_H_ from the Higuchi model for 0–5 h release vs. CNW content.

Assuming a diffusion-controlled release of tetracycline, the cumulative release curves were linearized according to the Higuchi model [35] (Figure 10a):

(4) $$Q=a+K_{H}\sqrt{t},$$

where Q is the cumulative tetracycline release (%); K_H_ is the Higuchi constant; t is the time (h).

The obtained curves were linear for 0–5 h. K_H_ is proportional to the drug diffusion coefficient in the matrix; therefore, the release within the first 5 h is prolonged due to limited diffusion, as K_H_ linearly decreases with increasing CNW content (Figure 10b). This limited diffusion is a result of increased gel viscosity and relaxation time (Figure 7). The parameters of the Higuchi model fitting are presented in Table 2. Release kinetics after the 5-h time point could not be correctly described with the Higuchi model due to significant swelling and a decrease in the tetracycline concentration. After 5 h, the rates of tetracycline release from the swollen ALG-CNW hydrogels were similar and independent from the CNW content (Figure 8).

## 4. Conclusions

Hydrogels were fabricated from ALG and partially deacetylated CNW. The ALG-CNW hydrogels were formed by various interactions between ALG and CNW polymer chains: electrostatic interactions upon the formation of PEC, entanglement of ALG chains, and hydrogen bonding. The strength of the ALG-CNW hydrogels depended on the number of CNW in the gel. The morphology of lyophilized hydrogels (layered for ALG and honeycomb for ALG-CNW) reflects the features of the structural organization of the hydrogels. For hydrogels, a more prolonged release of tetracycline was observed with an increased CNW content in the ALG hydrogel. Release curves correlated well with the Higuchi model. The mechanism of release prolongation most likely involves the modulation of tetracycline diffusion in the matrix. This diffusion can be controlled by manipulating the rheological properties of the gel (viscosity and relaxation time) through changes in the CNW content throughout the ALG hydrogel. The resulting hydrogels are biopolymers, and they formed simply by the intermolecular interactions of the polymers used, without the participation of crosslinking agents. These hydrogels may be of interest as soft gels for prolonged drug delivery.

## Figures and Tables

**Table 1:** Rheological properties of ALG-CNW hydrogels (***η_∞_*** = 0.0009 Pa∙s, *p* = 0.667).

Sample	CNW Mass Fraction, Relative Weight %	Test Mode	*τ_0_*, Pa	*η_0_*, Pa∙s	θ, s	Relative SD, %
ALG	0	Down SR	0.079	0.405	0.015	5.7
		Down F	20	–	–	–
		Top F	1120	–	–	–
		Top SR	435	–	–	–
ALG-CNW (2.5%)	2.5	Down SR	5.1	3.5	0.024	12
		Down F	1560	–	–	–
		Top F	900	–	–	–
ALG-CNW (7.5%)	7.5	Down SR	1.5	6.5	0.15	27
		Down F	2050	–	–	–
		Top F	18,000–44,000	–	–	–
		Top SR	3100	–	–	–
ALG-CNW (14.5%)	14.5	Down SR	14.4	114	3.4	1.5
		Down F	1820	–	–	–
		Top F	17,000	–	–	–
		Top SR	1450	–	–	–

**Table 2:** Fitting parameters of the Higuchi model for the tetracycline-containing ALG CNW hydrogels.

CNW Mass Fraction, Relative Weight %	I Phase (0–5 h)		
	R^2^	a	K_H_
0	0.989	−3.61	34.8
2.5	0.993	−6.79	33.3
7.5	0.988	−5.32	28.7
14.5	0.998	−10.0	23.7

## References

[1] Coviello T., Matricardi P., Marianecci C., Alhaique F. (2007). Polysaccharide hydrogels for modified release formulations. J. Control. Release.

[2] Dai T., Tanaka M., Huang Y.Y., Hamblin M.R. (2011). Chitosan preparations for wounds and burns: Antimicrobial and wound-healing effects. Expert Rev. Anti-Infect. Ther..

[3] Paul W., Sharma C.P. (2004). Chitosan and alginate wound dressings: A short review. Trends Biomater. Artif. Organs.

[4] Senni K., Pereira J., Gueniche F., Delbarre-Ladrat C., Sinquin C., Ratiskol J., Godeau G., Fischer A.M., Helley D., Colliec-Jouault S. (2011). Marine polysaccharides: A source of bioactive molecules for cell therapy and tissue engineering. Mar. Drugs.

[5] Kiroshka V.V., Petrova V.A., Chernyakov D.D., Bozhkova Y.O., Kiroshka K.V., Baklagina Y.G., Romanov D.P., Kremnev R.V., Skorik Y.A. (2017). Influence of chitosan-chitin nanofiber composites on cytoskeleton structure and the proliferation of rat bone marrow stromal cells. J. Mater. Sci. Mater. Med..

[6] Liu Z., Jiao Y., Wang Y., Zhou C., Zhang Z. (2008). Polysaccharides-based nanoparticles as drug delivery systems. Adv. Drug Deliv. Rev..

[7] Wu H., Williams G.R., Wu J., Wu J., Niu S., Li H., Wang H., Zhu L. (2018). Regenerated chitin fibers reinforced with bacterial cellulose nanocrystals as suture biomaterials. Carbohydr. Polym..

[8] Venkatesan J., Bhatnagar I., Manivasagan P., Kang K.H., Kim S.K. (2015). Alginate composites for bone tissue engineering: A review. Int. J. Biol. Macromol..

[9] Tønnesen H.H., Karlsen J. (2002). Alginate in drug delivery systems. Drug Dev. Ind. Pharm..

[10] Chávarri M., Marañón I., Ares R., Ibáñez F.C., Marzo F., del Carmen Villarán M. (2010). Microencapsulation of a probiotic and prebiotic in alginate-chitosan capsules improves survival in simulated gastro-intestinal conditions. Int. J. Food Microbiol..

[11] Ghidoni I., Chlapanidas T., Bucco M., Crovato F., Marazzi M., Vigo D., Torre M.L., Faustini M. (2008). Alginate cell encapsulation: New advances in reproduction and cartilage regenerative medicine. Cytotechnology.

[12] Draget K., Bræk G.S., Smidsrød O. (1994). Alginic acid gels: The effect of alginate chemical composition and molecular weight. Carbohydr. Polym..

[13] Grasdalen H., Larsen B., Smidsrød O. (1979). A PMR study of the composition and sequence of uronate residues in alginates. Carbohydr. Res..

[14] Shchipunov Y.A., Greben V., Postnova I. (2000). Preparation of calcium alginate gels by electrodialysis. Russ. J. Phys. Chem. A.

[15] Shchipunov Y.A., Koneva E., Postnova I. (2002). Homogeneous alginate gels: Phase behavior and rheological properties. Polym. Sci. Ser. A.

[16] Sæther H.V., Holme H.K., Maurstad G., Smidsrød O., Stokke B.T. (2008). Polyelectrolyte complex formation using alginate and chitosan. Carbohydr. Polym..

[17] Tam S., Bilodeau S., Dusseault J., Langlois G., Hallé J.P., Yahia L. (2011). Biocompatibility and physicochemical characteristics of alginate–polycation microcapsules. Acta Biomater..

[18] Etrych T., Leclercq L., Boustta M., Vert M. (2005). Polyelectrolyte complex formation and stability when mixing polyanions and polycations in salted media: A model study related to the case of body fluids. Eur. J. Pharm. Sci..

[19] Dragan E.S. (2014). Design and applications of interpenetrating polymer network hydrogels. A review. Chem. Eng. J..

[20] Kabanov V.A. (2005). Polyelectrolyte complexes in solution and in bulk. Russ. Chem. Rev..

[21] Khutoryanskiy V.V., Staikos G. (2009). Hydrogen-Bonded Interpolymer Complexes: Formation, Structure and Applications.

[22] Sarmento B., Ribeiro A., Veiga F., Sampaio P., Neufeld R., Ferreira D. (2007). Alginate/chitosan nanoparticles are effective for oral insulin delivery. Pharm. Res..

[23] George M., Abraham T.E. (2006). Polyionic hydrocolloids for the intestinal delivery of protein drugs: Alginate and chitosan—A review. J. Control. Release.

[24] Cook M.T., Tzortzis G., Khutoryanskiy V.V., Charalampopoulos D. (2013). Layer-by-layer coating of alginate matrices with chitosan–alginate for the improved survival and targeted delivery of probiotic bacteria after oral administration. J. Mater. Chem. B.

[25] Chang Y.H., Huang C.F., Hsu W.J., Chang F.C. (2007). Removal of hg^2+^ from aqueous solution using alginate gel containing chitosan. J. Appl. Polym. Ccience.

[26] Dai Q., Kadla J.F. (2009). Effect of nanofillers on carboxymethyl cellulose/hydroxyethyl cellulose hydrogels. J. Appl. Polym. Sci..

[27] Liu M., Huang J., Luo B., Zhou C. (2015). Tough and highly stretchable polyacrylamide nanocomposite hydrogels with chitin nanocrystals. Int. J. Biol. Macromol..

[28] Yang X., Bakaic E., Hoare T., Cranston E.D. (2013). Injectable polysaccharide hydrogels reinforced with cellulose nanocrystals: Morphology, rheology, degradation, and cytotoxicity. Biomacromolecules.

[29] Zhang X., Huang J., Chang P.R., Li J., Chen Y., Wang D., Yu J., Chen J. (2010). Structure and properties of polysaccharide nanocrystal-doped supramolecular hydrogels based on cyclodextrin inclusion. Polymer.

[30] Lin N., Huang J., Chang P.R., Feng L., Yu J. (2011). Effect of polysaccharide nanocrystals on structure, properties, and drug release kinetics of alginate-based microspheres. Colloids Surf. B Biointerfaces.

[31] Petrova V.A., Panevin A.A., Zhuravskii S.G., Gasilova E.R., Vlasova E.N., Romanov D.P., Poshina D.N., Skorik Y.A. (2018). Preparation of N-succinyl-chitin nanoparticles and their applications in otoneurological pathology. Int. J. Biol. Macromol..

[32] Bajpai A.K., Shukla S.K., Bhanu S., Kankane S. (2008). Responsive polymers in controlled drug delivery. Prog. Polym. Sci..

[33] Bardajee G.R., Pourjavadi A., Soleyman R. (2011). Novel nano-porous hydrogel as a carrier matrix for oral delivery of tetracycline hydrochloride. Colloids Surf. A Physicochem. Eng. Asp..

[34] Ueng S.W., Lee M.S., Lin S.S., Chan E.C., Liu S.J. (2007). Development of a biodegradable alginate carrier system for antibiotics and bone cells. J. Orthop. Res..

[35] Siepmann J., Peppas N.A. (2011). Higuchi equation: Derivation, applications, use and misuse. Int. J. Pharm..

